# Factors affecting patient recruitment to trials: qualitative research in general practice

**DOI:** 10.3399/bjgpopen20X101056

**Published:** 2020-08-19

**Authors:** Marian J van den Brink, Monique Hummel, Marrit Lemstra, Marjolein Y Berger, Janny H Dekker, Marco H Blanker

**Affiliations:** 1 Department of General Practice and Elderly Care Medicine, University of Groningen, University Medical Centre Groningen (UMCG), Groningen, The Netherlands; 2 Department of Human Movement Sciences, University of Groningen, University Medical Centre Groningen (UMCG), Groningen, The Netherlands

**Keywords:** primary health care, qualitative research, randomised controlled trial, general practice

## Abstract

**Background:**

Patient recruitment to clinical research is often challenging and, when inadequate, can result in delayed or underpowered studies. Recruitment problems were experienced during a study of women with heavy menstrual bleeding in general practice (the MIRA trial). Although efforts were made to reduce the burden of the study for those participating, patient recruitment was still an issue.

**Aim:**

To identify the barriers and facilitators associated with patient recruitment to clinical trials, as experienced by GPs.

**Design & setting:**

A qualitative study was performed in Dutch general practice, using semi-structured interviews.

**Method:**

GPs participating in the MIRA trial were selected by purposive sampling and interviewed until saturation was reached. Three independent researchers performed data coding and thematic analysis. Consensus on the identified themes was reached by discussion among the researchers.

**Results:**

Sixteen GPs were interviewed. The following factors were noted to influence recruitment: the incidence of the disease under study; awareness of the study; attitude towards scientific research; perceived burden for the patient; usual care by the GP; time investment; characteristics of the GP and their practice; and patient experience of research participation.

**Conclusion:**

The identified barriers and facilitators associated with patient recruitment highlight the areas in which future studies can be improved. Indeed, benefits could be gained by simply ensuring that study procedures are clear, by requiring limited (time) investment from the GP, and by investing in personal communication and reminders to keep the GP motivated and interested. Placing greater importance on scientific research during the GP training programme could also serve as a means to motivate future GPs to integrate scientific research in their clinical practice.

## How this fits in

Enrolling a sufficient number of patients to a clinical trial can be difficult, but there have been few studies into recruitment problems in primary care. This qualitative study identified the barriers and facilitators associated with patient recruitment, as experienced by GPs. Disease incidence, study awareness, attitudes to research, patient burden, usual GP care, time investment, GP and practice characteristics, and patient experience were all found to be relevant. It is concluded that future studies may be more successful if researchers take these factors into account.

## Introduction

Randomised controlled trials (RCTs) are a powerful research tool that can minimise bias when evaluating health interventions and help to deliver evidence that is of high quality.^1^ However, recruitment delays and issues (clinician and patient) continue to plague trials, significantly impacting costs and investigator workloads. Failure to enrol adequate numbers can adversely affect the statistical power of a study and the value of any results.^2^

Research in general practice has expanded in recent decades. In most primary care studies, patient recruitment often takes more time than expected. This phenomenon is known as Lasagna’s Law: the number of patients that can actually be recruited during research is only a fraction of that estimated by researchers based on incidence data from morbidity registries.^3^ For example, in a survey of 78 studies in Dutch general practice, fewer than 50% of the researchers recruited the required number of patients within the planned schedule. Studies recruiting incident cases face larger problems with patient recruitment than studies identifying cases via GP registration systems (prevalent cases).^4^ The inclusion of incident cases requires that GPs are alert to patient recruitment during consultation hours, which can prove challenging. Studies on this topic have reported large variations in GP recruitment of patients.

The reasons for the differences in recruitment success among GPs remain unclear because most research into recruitment problems has focused on secondary or tertiary care, and, as such, has failed to consider the perspectives of GPs.^2,5^ Major problems were encountered with patient recruitment in a multicentre RCT conducted in the Netherlands, which sought to compare the levonorgestrel-releasing intrauterine system (LNG-IUS) and endometrial ablation for the treatment of heavy menstrual bleeding (HMB), the MIRA trial.^6^ Patients were recruited by GPs or gynaecologists, required to give their consent for randomisation to a study arm, and asked to complete questionnaires at baseline and at four follow-up points. Despite making reasonable efforts to reduce the burden of the study for participating GPs and patients, patient recruitment was problematic. It has been noted that failure to recruit patients for prospective studies can occur at different stages of the recruitment process: stage one concerns obtaining agreement from a clinician to participate; stage two concerns the actual recruitment of patients; stage three concerns obtaining the agreement of a patient to participate; and stage four concerns the agreement of the patient to remain in the study.^7^

This qualitative study aimed to identify the facilitators and barriers associated with successful patient recruitment by GPs in clinical trials. The study focused on stages two and three of the recruitment process.

## Method

### Study design

A qualitative study was performed, using semi-structured interviews, to identify the barriers and facilitators associated with GP recruitment of patients. The Consolidated Criteria for Reporting Qualitative Research (COREQ) and the Standards for Reporting Qualitative Research (SRQR) were applied.^8,9^

### Study population

GPs were selected who had participated in the MIRA trial^6^ using purposive sampling to obtain the maximum variety of themes that influence patient recruitment. Sampling was based on sex, age, practice type (solo or group), practice location (urban or rural), and number of patients recruited for the MIRA trial. Interviews continued until data saturation occurred.^10^

### Semi-structured interviews

The semi-structured interviews were based on a pre-specified interview guide that sought to gain as many personal insights as possible. The interview guide was based on existing literature and was developed through discussion within the research group.^5,11–13^ Included topics were: the reasons for participation in scientific research, communication between GP and researcher, and communication with patients about participation in clinical research. In all instances, GPs were asked to consider the question both in general and with reference to the MIRA trial. Therefore, the guide also included questions about knowledge of the MIRA trial and experience with, and treatment preferences for, HMB.

A sixth-year medical student (MH), who was not involved in the MIRA trial, performed the face-to-face interviews between February and April 2015. She received individual training in communication and interview techniques, consisting of multiple sessions with a GP specialised in qualitative interviewing. Afterwards, her skills were assessed in a pilot interview with a GP. This pilot interview was videotaped and evaluated by members of the research group to improve the interview technique and guide. GPs were invited by email and telephone, and informed about the objectives and topics as well as the anonymity of their responses before the interview.

### Data handling and analysis

All interviews were tape recorded, transcribed verbatim, and anonymised. A summary of the interview was sent to each participant for response validation. Three researchers independently performed data coding and thematic analysis, using the ATLAS.ti software (version 7.5). These researchers had different backgrounds, enabling them to look at the topics from different angles to foster a reflexive discussion and to enhance the reliability of the results. At the time of the interviews, MJB was a GP trainee and researcher for the MIRA trial, and MH was a sixth-year medical student. ML has a master’s degree in human movement sciences.

Data coding was performed iteratively in parallel with the interviews. During this process, the researchers (MJB, MH, and ML) discussed the independently identified codes and categorised them into themes. Discrepancies in factors and themes, as well as the overview of themes with their mutual relationship, were discussed in the entire research group until consensus was reached. In the text, each theme is illustrated with quotes from individual GPs, to which their age and sex have been added, as well as whether they recruited patients. The quotes were translated from Dutch to English by a certified translator.

## Results

### Interviews

Twenty GPs were invited, of whom four were unavailable owing to lack of time, leaving 16 who agreed to participate. The characteristics of the 16 participants are presented in Table 1 (eight females and eight males, aged 38–61 years). Of these, six included patients in the MIRA trial and 10 consented to participate in the MIRA trial but did not include patients. The interviews lasted 32 minutes on average and themes were saturated after 12 interviews. When interviewing the remaining four GPs, no new themes emerged.

**Table 1.** Characteristics of participating GPs, *N* = 16

**Characteristics**	***n* (%)^a^**
**Sex** **, male**	8 (50)
**Median age** **,** **years (range)**	52 (38–61)
**Median GP experience** **,** **years (range)**	19 (5–35)
**Competent in LNG-IUS insertion**YesNo	15 (94)1 (6)
**Median patients per GP** **,** ***n*** **^b^ (range)**	2170 (1600–3650)
**Type of general practice**Solo practiceDuo practiceGroup practice	10 (63)^c^3 (19)3 (19)
**Location** **of** **general practice**UrbanRuralMedian distance to nearest participating hospital, km (range)	9 (56)7 (44)5.9 (0.9–25.4)
**R** **ecruited patients for MIRA trial** **,** ***n***0≥1	10 (63)6 (38)
**Other characteristics**GP educatorDoctorate degreePharmacy owner combined with GP	4 (25)3 (19)1 (6)

^a^Unless otherwise indicated. ^b^Number of patients registered per full-time working GP. The standard number of patients registered in a general practice in the Netherlands was 2168 patients in 2014.^25^ ^c^Including eight GPs who owned a private practice but shared a building with other GPs. LNG-IUS = levonorgestrel-releasing intrauterine system.

### Themes related to patient recruitment

**Figure 1.**
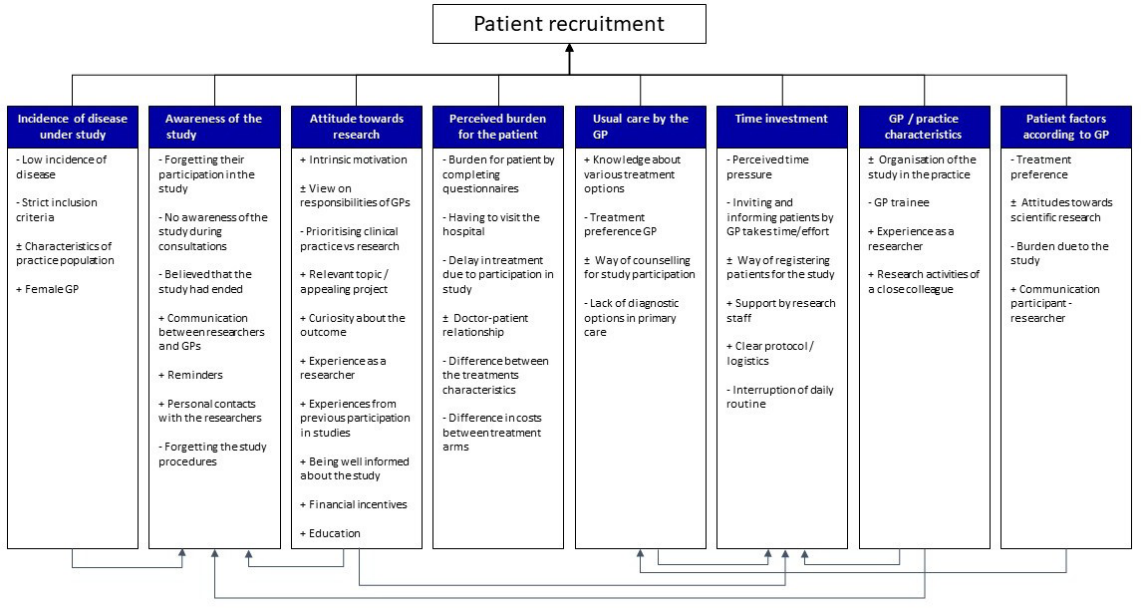
Factors related to patient recruitment for scientific research in general practice. The eight themes and their corresponding factors are shown with an indication as to whether the factor could positively (+) or negatively (-) influence recruitment (± if both were possible). Relevant connections between themes are shown by the ligatures at the base of the image.

The factors related to patient recruitment were categorised into eight themes by the three researchers involved in coding, with each theme noted to have been discussed by both GPs who recruited and who did not recruit patients. The eight themes and their corresponding factors are shown in Figure 1.

#### Incidence of the disease under study

Many participants mentioned the low incidence of the condition studied in the trial (that is, HMB) as a barrier to patient recruitment, and some commented that the incidence also depended on the practice characteristics:

*‘My practice is in the city centre, so I get to see mainly students, young students, etc. Plus families with children, as well as the elderly. And then rather more of the elderly, and hardly anyone in between. So maybe that also plays a role: that the pond I’m fishing in isn’t that big, if you like.’* (Male, aged 58 years, non-includer)

The strict inclusion criteria required for the MIRA trial further hampered recruitment and were often mentioned as a barrier for patient recruitment in general. Female GPs did mention seeing more women with HMB because many women preferred seeing a female GP for a gynaecological problem.

#### Awareness of the study

The theme 'low incidence of the disease' was related to the theme ‘awareness’. One barrier that was mentioned often by GPs was that they forgot that they were participating in the trial and did not remember the study when consulting an eligible patient. Some GPs mentioned that they thought that the trial had already ended:

*‘And then at some point, you lose interest, especially when you don’t hear anything over a period of time and don’t get triggered; then it will disappear to the background, and eventually you forget about it.* […] *Yes, you forget and then you ask yourself why that happened, and you put it down to being too busy, having other priorities, other things, then this kind of thing just quickly recedes to the background.’* (Male, aged 38 years, non-includer)

A lack of readily available information about the study procedures was also mentioned as a barrier to recruitment:

*‘Then I first have to look and see if they are eligible* [leafs through information forms of the MIRA study] *and yes, then I don’t know, and then I just leave it.’* (Female, aged 59 years, non-includer)

Communication between researcher and GP was commonly mentioned. GPs indicated that reminders or personal contact with the researchers motivated them to recruit patients. However, they gave no uniform response about the form of reminder that would be most appreciated; for example, suggestions included a newsletter, feedback about the number of included patients, and telephone contact with the possibility to ask questions.

#### Attitude towards scientific research

The GP attitude towards scientific research was considered important not only for their commitment to participate, but also for their commitment to recruit patients. GPs who liked to participate in scientific research had less trouble inviting patients, whereas others felt that daily practice and scientific research were difficult to combine. Some GPs also mentioned that participating in research was a responsibility that came with the job:

*‘For some reason, research and patient care don’t seem to go together, and that’s frustrating, because you know that you need each other and yet they just don’t seem to fit.’* (Male, aged 53 years, includer)

*‘And of course, there’s the obligation on the part of GPs to recruit patients. Otherwise you can’t externalise … generalise the results.’* (Male, aged 53 years, includer)

Factors that facilitated GP participation and willingness to invest time in a study included the following: a relevant research question; a research project specific to GPs; curiosity about the study outcomes; and having experience with research. The latter of these included personal experience as a researcher and experience with patient recruitment in other studies. The extent to which a GP had studied the research topic and the study procedures also affected recruitment (see ‘awareness of the study’). Finally, GPs mentioned that incentives for participation would be welcomed. This may include financial incentives for recruiting patients, acknowledging the time investment; however, it may also include educational incentives based on recognition that participating in a study increases knowledge of the topic and has inherent educational value.

#### Patient burden, as perceived by the GP

The burden placed on the patient when participating in a study was generally acknowledged as a barrier to recruitment. This expected burden was reinforced by the marked difference in treatments between the two study arms. Participation in the study could lead to financial costs, an increased burden from hospital visits and possible treatment delay. In the MIRA trial, the invasiveness of endometrial ablation and the need to refer women to the hospital when randomised to endometrial ablation led to many GPs preferring the LNG-IUS. Thus, some GPs reported that they either did not invite women or that they invited them selectively:

*‘And definitely now that people have to pay their deductible,^a^ which they probably won’t have to spend because they’re often still pretty young. So those are the considerations and they’re all things that you have to explain to the patients.’* (Female, aged 38 years, non-includer)

^a^In the Netherlands it is mandatory to take out health insurance for basic care, with a deductible (€385 in 2020) applicable to certain healthcare costs. Notably, GP care is exempt.

Some GPs mentioned that a good doctor–patient relationship serves as a barrier to patient recruitment, whereas others stated that a good relationship could facilitate recruitment:

*‘I do think that it also has something to do with the fact that you’ve built up a good personal relationship with the patient and that can prove to be a stumbling block; that you get the idea that you’ll be putting the people under extra pressure. I think that’s how it works, that’s the way it is. And it’s not something you can resolve.’* (Female, aged 59 years, non-includer)

#### Usual care by the GP

The extent to which different treatment options for HMB were known and used by the GP in daily practice was important when enrolling patients to the MIRA trial. Some GPs preferred drug treatment over the LNG-IUS or ablation, and some mentioned lack of knowledge about ablation:

*‘Of course, we only insert the Mirena, so that’s why we know it’s good. And, well, to be honest, I don’t really know that much about ablation.’* (Female, aged 55 years, non-includer)

Many GPs participating in the MIRA trial favoured the LNG-IUS over ablation, which hampered patient recruitment. They commented that it was reversible and easy to provide in general practice, making it preferable to hospital referral. Other GPs preferred ablation therapy:

*‘They’re really fundamentally different treatments you know. One is for the rest of your life, and the other one is temporary.’* (Male, aged 58 years, includer)

In some cases financial considerations also played a role because LNG-IUS insertion generates extra income. However, patient experience strongly guided GP preference for or against a treatment option. That said, approaches to counselling patients for study participation differed markedly among GPs: some applied a paternalistic approach, advocating participation, while others informed patients about the study and made participation a shared decision.

Some GPs stated that they did not recruit patients if they wanted more diagnostic certainty beforehand, and that they would refer to a gynaecologist for diagnosis and treatment if they lacked the facilities to screen patients by vaginal ultrasound to exclude uterine abnormalities.

#### Time investment

Nearly all GPs mentioned that the investment of time and effort was a barrier to ensuring patient recruitment. In general, GPs mentioned that they experienced time pressure related to clinical practice, administration, management duties, or having a pharmacy connected to their practice. Consequently, the limited 10-minute consultations were deemed too short to allow for both clinical consultation and active patient recruitment. Some GPs mentioned that this was the main reason for not approaching patients about the study, but others did suggest that it may be reasonable to reschedule an appointment or invite the patient at another time:

*‘But really, you’re under pressure throughout your office hours. And usually by the end of the morning I’m running 20–30 minutes late. And if I’m unlucky, even an hour, with a lot of urgent cases coming in unexpectedly sometimes. So yes, it soon becomes too much work, that’s what it comes down to.’* (Female, aged 59 years, non-includer)

Interestingly, when both treatment options were familiar to the GP, this influenced the decision to invest time irrespective of the time constraints:

*‘Of course, if someone comes to you with symptoms and there’s nothing wrong, you have to explain what the options are, so the research doesn’t really mean a lot more work.* […] *So in theory all you have to do is mention the research, including the options; and yes that means a few sentences extra, but it’s not that much more work; it shouldn’t be that much of a problem.’* (Female, aged 49 years, non-includer)

Patient recruitment to research was perceived as an interruption of the daily routine:

*‘The difficult part is stepping out of your routine, because you always do certain things in a specific way, these are patterns that at some point have become automatisms. And at some point, you have to be made aware of this, and start to think “we could do this differently”.’* (Male, aged 53 years, includer)

Time investment seemed to be related to the theme ‘attitude towards scientific research’. The perceived relevance of the research question and topic increased the willingness of GPs to invest time for a study. One participant (male, aged 60 years, non-includer) suggested that it was the low priority to include patients for a given study and not the time constraints that was the main factor affecting engagement.

#### GP and practice characteristics

The way research projects were organised in practices was important to GPs. Potential facilitators were to ensure a key role for the practice assistant and to plan extra time allowances for when a potential participant was scheduled:

*‘That pile of papers is somewhere here, but more and more stuff keeps getting put on top of it, if you see what I mean. I need to search through the whole pile if I want to give patients something to take with them.’* (Female, aged 48 years, includer)

One participating GP (male, aged 58 years, includer), also a GP trainer, mentioned that it was difficult to get trainees involved in research and that this may also lower recruitment. GPs with personal experience of research (for example, having a PhD) and those with a close colleague involved in research had a better understanding of the importance of patient recruitment:

*‘If there’s just one person who’s enthusiastic about it, and who tries to get the others interested* [via the HAGRO^b^]*. In fact, it’s like word of mouth, that you know someone who’s enthusiastic about it. We’ve got a few GP trainers in our group who are linked to the university general practitioners training, and via them you’re more likely to come into contact with these kinds of issues.’* (Female, aged 39 years, non-includer)

^b^HAGRO: group of 5–12 GPs working in the same area and acting as a peer group.

#### Patient factors according to the GP

Treatment preference mentioned by the patient was perceived by the GPs to be an important barrier and reason for non-participation in the MIRA trial. GPs felt that the attitudes of patients toward scientific research were generally positive, but that the expected burden of participation in a study could be a barrier, commenting that some patients were tired of research participation:

*‘In general, people were very open about participating, and sympathetic, so that was a positive experience for me.’* (Female, aged 38 years, includer)

*'Yes, really tired of research, I clearly notice that. So I said for this year that I am not going to do any new research.* [...] *People also know that they are in a research practice. And they will be asked more often, but they have also learned to say no. But I think that's the biggest bump, asking too much for research.'* (Male, aged 60 years, non-includer)

Good communication between the researcher and patients who were already participating was considered a facilitator of further patient recruitment. Unfortunately, some patients had told their GP that they had missed the feedback from researchers about their questionnaire responses.

## Discussion

### Summary

This was a qualitative study among GPs about the barriers and facilitators associated with patient recruitment for clinical trials, with a focus on experiences in the MIRA trial. Important barriers to recruiting patients were the need to invest extra time, the necessary interruption to daily practice, poor awareness of the study because of the low incidence of the disease under study, GP preference for one of the study treatments, and the perceived burden for the patient. By contrast, the main facilitators were a positive attitude towards scientific research, a relevant topic, a clear study design, study procedures that required limited time investment by the GP, personal communication, and reminders tailored to the individual GP.

### Strengths and limitations

Rather than interviewing researchers or patients, it was decided to interview GPs about their experiences of, and attitudes to, recruitment in primary care. It was anticipated that this approach would provide a unique qualitative analysis of the barriers and facilitators associated with patient recruitment from the perspective of GPs, which is important because the GP is a key link in the recruitment of incident patients for clinical trials in primary care. This study benefitted from being able to question GPs using a concrete example of a trial in which they participated, avoiding the need for purely hypothetical questions. However, although GPs were interviewed who did and did not recruit patients, all the interviewed participants had agreed to recruit patients for the clinical trial. This may mean that the researchers spoke preferentially to GPs who were more positive about participating in scientific research than their peers. All the GPs were involved with the MIRA trial, which might have led to the authors missing themes or factors arising from other studies. Also, asking patients their opinion could have revealed more patient-related factors, but the goal was to ascertain the perspective of GPs only.

### Comparison with existing literature

To date, most studies about factors affecting patient recruitment in clinical trials have been conducted in secondary care, with relatively few in primary care settings.^14,15^ It is known that reduced awareness of a study can cause recruitment problems and that reminders are a meaningful addition to a study protocol. Approaches may include a practice visit by the researcher or the sending of printed educational material to the practice.^14,16^ The responders in the present study highlighted that personal communication was an important factor. However, this is time consuming for both researchers and GPs, and the different responses in the interviews indicated that there was no universally preferred approach.

There is an established association between patient preference for a particular treatment and difficulties with recruitment.^2,17^ Patient preference is more common in trials where there is a large difference between the characteristics of the two treatment arms. Although patient preference was not investigated directly, it is known that it is affected by whether a GP gives neutral counselling.^12^ Research has shown that GPs may be selective in asking patients to participate in studies because of both patient characteristics and their own preferences for a given treatment.^17,18^ This was confirmed by many GPs in this research.

A factor relevant to the primary care setting was the attitude of the GP towards scientific research. Combining clinical care, education and research is much more common in (academic) hospitals. In primary care, however, clinical care and patient welfare seem to have a higher priority, with evidence that scientific research is viewed as having too little intrinsic, professional, or clinical value.^2,5,19,20^ This theme also emerged in the interviews. Although lack of time appeared to be a major barrier, this was highly dependent on a given GP's priorities and responsibilities.

Foster *et al* reported that poor recruitment was associated with a longer time until the first patient was included in the study, poor access to potential patients, and working in a training practice. Support by research staff could increase recruitment rates, with the long-term support possibly leading to improved recruitment skills among GPs.^21^

### Implications for research and practice

The results of this study raise several possibilities for integrating clinical practice and research in primary care. Research participation should be a task required of all GPs, and it should be a matter of course that they cooperate in research and contribute to evidence-based care in general practice. Participation could then be better valued with accreditation points or education. The Dutch Association of General Practice endorses this importance in their vision document, in which they state that the profession should take responsibility for the continuity and development of the discipline, with every general practice participating structurally in education, research, or innovation.^22^

Attention is needed to encourage the participation of GP trainees in research and to produce practical recommendations on how to combine practice and research. Over time, it is hoped that these approaches will bridge the gap between research and clinical practice among GPs. Specific attention should be paid to recruitment skills and to how to deal with situations where the GP prefers one study arm or is more familiar with one treatment option. However, researchers must also ensure that there is a clear study protocol that requires as few extra tasks as possible for the GP, possibly including financial compensation for the additional time investment. However, given the available evidence, financial reimbursement alone would only contribute minimally to patient recruitment, so other options must be considered.^23^ Patients and GPs should be involved in the design of a study to advise on important issues, such as relevance of the research question, outcome measures, and feasibility of the study.

This study also revealed factors that cannot be influenced by researchers, such as conditions with a low incidence in general practice. This situation requires large numbers of recruiting GPs and additional efforts to ensure awareness of the study during the inclusion period. Involving administrative staff or nurses in the general practice or innovations in information technology (for instance, pop-ups in GP registration systems) may contribute to raising awareness and enrolment. Research departments could also facilitate practices by training employees to inform patients about studies, and where appropriate, recruit suitable candidates.
